# Power provides protection: Genetic robustness in yeast depends on the capacity to generate energy

**DOI:** 10.1371/journal.pgen.1006768

**Published:** 2017-05-11

**Authors:** Marcin Plech, Katarzyna Tomala, Hanna Tutaj, Dominika Ewa Piwcewicz, J. Arjan G. M. de Visser, Ryszard Korona

**Affiliations:** 1 Institute of Environmental Sciences, Jagiellonian University, Krakow, Poland; 2 Laboratory of Genetics, Wageningen University, HB Wageningen, Netherlands; University of Pennsylvania, UNITED STATES

## Abstract

The functional basis of genetic robustness, the ability of organisms to suppress the effects of mutations, remains incompletely understood. We exposed a set of 15 strains of *Saccharomyces cerevisiae* form diverse environments to increasing doses of the chemical mutagen EMS. The number of the resulting random mutations was similar for all tested strains. However, there were differences in immediate mortality after the mutagenic treatment and in defective growth of survivors. An analysis of gene expression revealed that immediate mortality was lowest in strains with lowest expression of transmembrane proteins, which are rich in thiol groups and thus vulnerable to EMS. A signal of genuine genetic robustness was detected for the other trait, the ability to grow well despite bearing non-lethal mutations. Increased tolerance of such mutations correlated with high expression of genes responsible for the oxidative energy metabolism, suggesting that the negative effect of mutations can be buffered if enough energy is available. We confirmed this finding in three additional tests of the ability to grow on (i) fermentable or non-fermentable sources of carbon, (ii) under chemical inhibition of the electron transport chain and (iii) during overexpression of its key component, cytochrome c. Our results add the capacity to generate energy as a general mechanism of genetic robustness.

## Author summary

The ability to suppress phenotypic effects of mutations is termed genetic robustness. Its functional basis and evolutionary origin remain insufficiently understood despite decades of research. In fact, it is still largely untested whether genetic robustness is a trait of substantial, within-species variation. We used a model organism, *Saccharomyces cerevisiae*, to study both phenotypic signs and functional underpinnings of genetic robustness. We introduced random mutations into a set of well-characterized yeast strain. There was considerable variation in the growth rate among clones recovered after mutagenesis, which is an indication of genetic robustness. Using available data on gene expression for our strains, we found that genetic robustness was strongest among strains with enhanced expression of genes related to the energy metabolism. We reasoned that, regardless of the specific mutations, the capacity to generate metabolic energy may be a general underlying mechanism for buffering the effects of random mutations across the genome. We confirmed this hypothesis in further experiments in which we showed that genetic robustness decreases when the energy metabolism is compromised and increases when it is boosted.

## Introduction

Robustness is the ability of an organism to perform its functions when faced with genetic or environmental perturbations [1]. Robustness against mutations is particularly intriguing. Numerous studies have found that a surprisingly large proportion of gene-inactivating mutations have little consequence for fitness, both in microorganisms and metazoans [2]. In the budding yeast, knockouts of single genes showed that only about one fifth of them are essential for growth while two fifths have effects undetectable under standard laboratory conditions [3, 4]. The simplest explanations point to functional redundancy as the source of genetic robustness. However, even in the budding yeast, a species that underwent a whole genome duplication event early in its history [5], the ubiquity of dispensable genes can be only partly explained by the presence of their duplicates [6–8]. Other hypotheses say that genetic robustness may also be a non-selected byproduct of other traits [9–12] or a feature evolved in congruence with environmental robustness [13–15]. It has been also suggested that genetic robustness can be an autonomous trait that evolved not only to help in current functioning of organisms but also to facilitate their evolvability [10, 16–18].

Robustness can be rooted in many processes and features [19, 20]. However, some elements of the eukaryotic cell are more likely than others to be essential in the face of perturbation. Examples include enzymes involved in mRNA processing, protein quality control and chaperoning, protein modifications and chromatin remodeling [21]. The best known among them are molecular chaperones, a class of proteins engaged in primary folding and refolding of destabilized proteins [18, 22, 23]. Indeed, overexpression of the GroEL chaperone is likely to mitigate the effects of mutational load accumulated under genetic drift in bacteria [24, 25]. However, chaperones also act as examiners of proteins, often triggering their degradation and depletion [26, 27], and therefore their overall impact on masking or exposing mutations has to be carefully examined [28, 29]. Another group of molecules putatively involved in robustness are chromatin modulators [30, 31]. They may help to hide genetic variation by condensing chromatin in some regions of the genome, to later release this variation when conditions change [32].

We asked whether a set of diverse strains of the budding yeast *Saccharomyces cerevisiae* exhibit differences in their ability to tolerate random mutations. These strains come from a collection of wild and domesticated strains that has been analyzed extensively [33–40]. In particular, complete genome sequences are available, as well as transcript abundances determined under controlled laboratory conditions [34]. We mutagenized a subset of 15 of these strains with increasing doses of ethyl methanesulfonate (EMS). The treatment resulted in similar numbers of mutations, but the phenotypic response to mutagenesis varied considerably, indicating variation in genetic robustness. Analysis of mRNA expression data showed that there was a strong correlation between genetic robustness and the ability to generate metabolic energy. This finding was then confirmed experimentally in three different tests: a comparison of growth on fermentable and non-fermentable carbon sources, effects of chemical inhibition of respiration and effects of overexpression of an element of the electron transport chain.

## Results

### Mutation rate at the *URA3* locus is uniform across all strains

**Fig 1:**
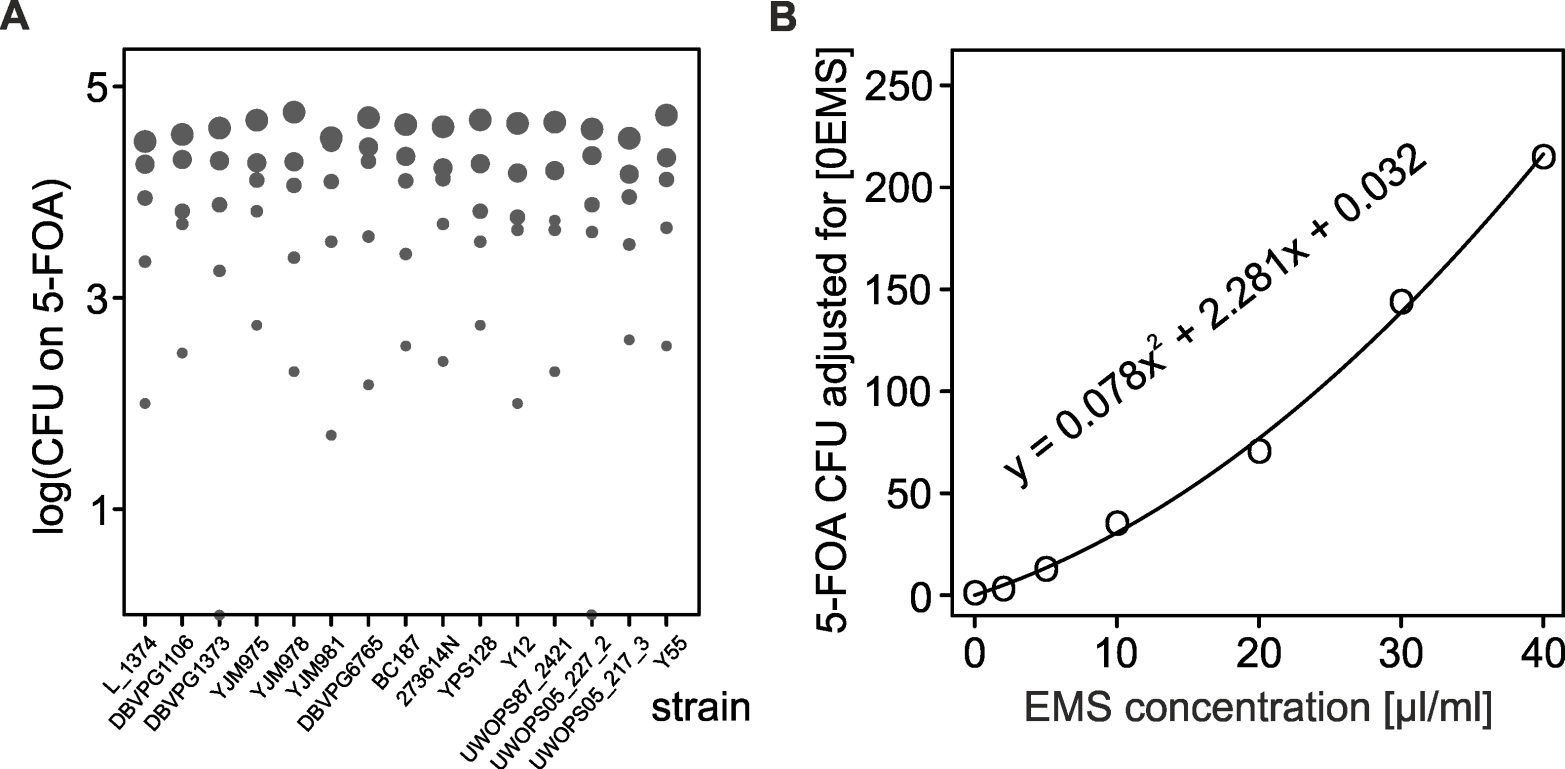
Relationship between EMS dosage and numbers of mutations introduced. (A) Number of *URA3* mutations per strain per concentration estimated from c.f.u. counts on medium containing 5-FOA. The increasing sizes of dots represent increasing doses of mutagen, from 0 to 30 μl/ml. (B) The relation between the dose of EMS applied and the mutant count approximated by a polynomial function. The function is then used to replace the EMS dose by the “relative mutation load” measuring the increase in mutation number relative to untreated cells. (There are seven EMS doses at B and only five in A because for two doses, 2 and 40 μl/ml, there were lacking replicas and they were excluded from ANOVA testing for the strain and dose effects).

Strains used in this study derive from a well-characterized collection of wild and domesticated strains of *S*. *saccharomyces* (Liti et al. 2009). They were originally deleted for *URA3* [41]. We re-introduced a functional copy of this gene, so that it could be used as a reporter of inactivating mutations. Clones carrying such mutations are able to grow on media containing 5-fluoroorotic acid (5-FOA). We first asked whether individual strains acquire mutations at similar rates when treated with EMS. The strains were exposed to a range of EMS doses up to ones typically used in mutagenesis experiments (30 or 40 μl/ml). EMS is known to introduce mainly single base-pair substitutions [42], a class of mutations known to prevail also among spontaneous mutations inactivating the target gene, *URA3* [43]. Fig 1A shows that the number of mutants increased significantly with the increasing dose of mutagen (*F* = 673.5; dfs = 4, 74; *p* < 0.0001). Crucially, there were no differences between strains in the number of mutants (*F* = 1.4; dfs = 14, 74; *p* = 0.162). We, therefore, could average the frequencies of mutations across strains and fit a quadratic function, one for all strains, describing the relationship between the dose of EMS and the number of mutations it introduced (Fig 1B). (Data needed for this analysis and graph, as well as all other analyses and graphs, are shown in S1 Table in the Supporting Information).

### Mortality is explained by the toxic effect of EMS

**Fig 2:**
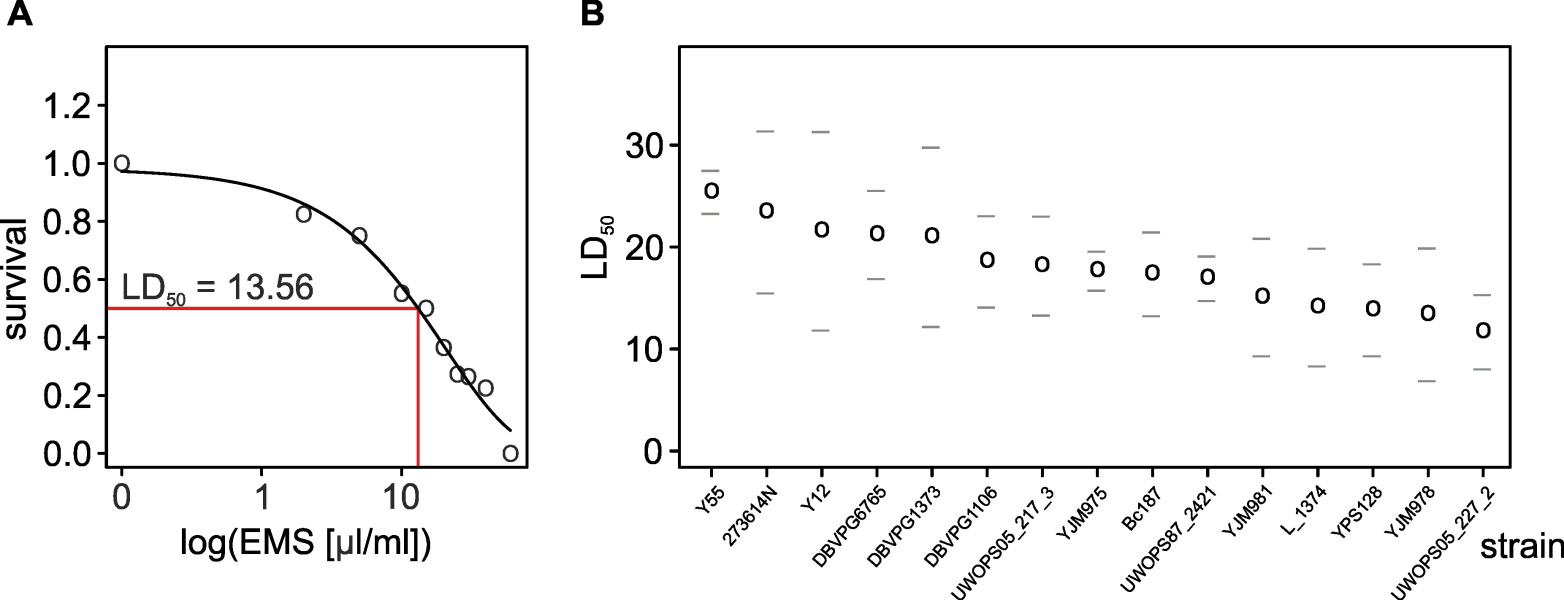
Survival immediately after EMS treatment. (A) Example of a single strain’s survival curve. A Weibull curve is fitted to estimate the LD_50_ (lethal dose killing 50% of population). Each point is the mean of two replicates. (B) LD_50_ values with 95% confidence limits of individual strains ranked according to EMS sensitivity (low sensitivity requires a high dose of EMS for LD_50_).

These results mean that the frequency of mutations at the molecular level increased with an increasing dose of EMS at a similar rate in all compared strains. We then asked whether the same was true for the rate of mortality. As expected, the number of survivors differed between doses of the mutagen (*F* = 913.2; df = 9; 74; *p* < 0.0001). Unlike the rate of mutation to Uraˉ, the rate of mortality differed between individual strains (*F* = 9.62; df = 14, 74; *p* < 0.001). To further test for differences between strains, we fitted the data to survival curves (the Weibull survivor function performed best when compared with log-logistic and log-normal models) and calculated for individual strains the EMS dose which was lethal for 50% of exposed cells (LD_50_). Fig 2 shows that the LD_50_ of the most sensitive strains was about twice as low as that of the most resistant ones.

**Fig 3:**
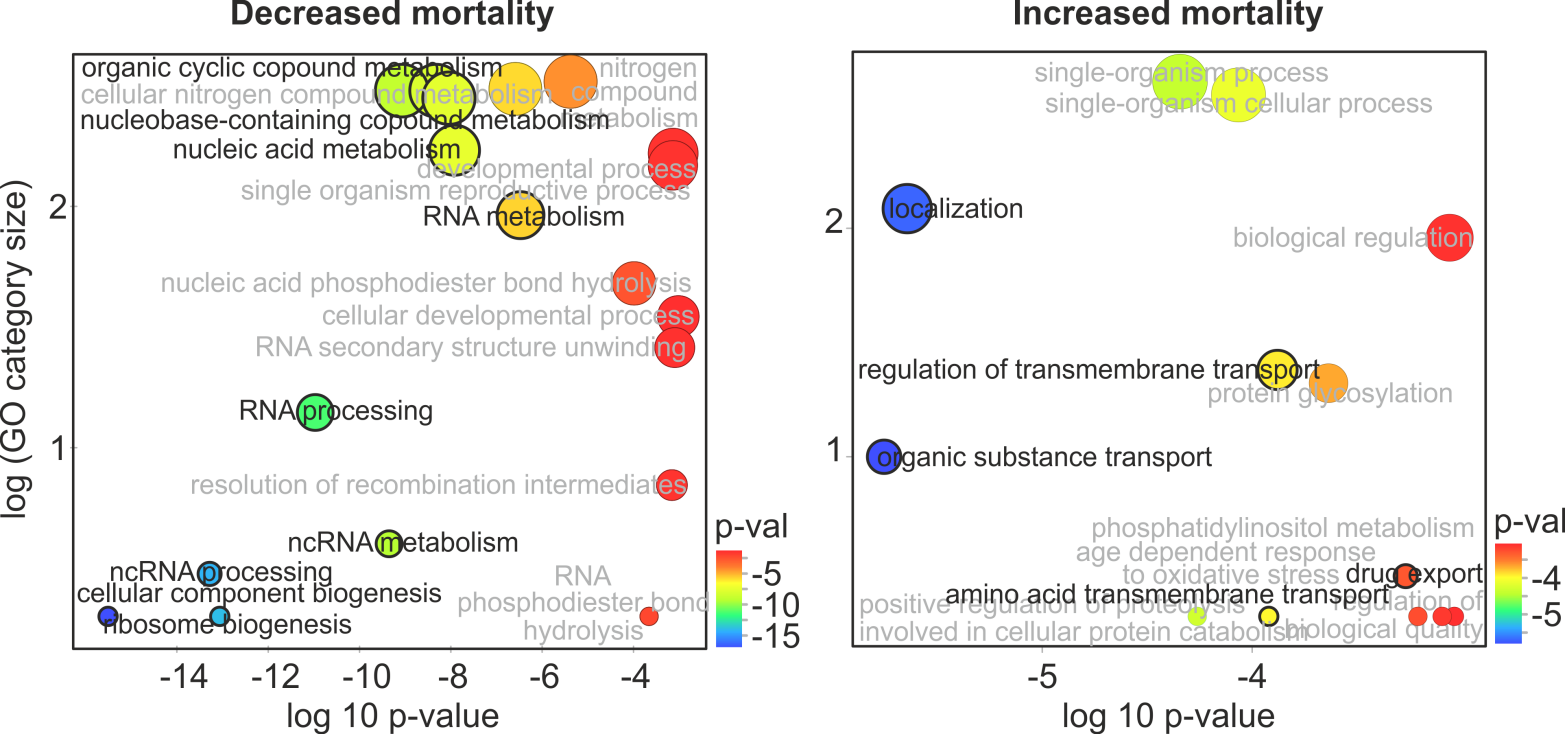
Test for functional determinants of mortality immediately after EMS treatment. Enhanced expression of mRNA transcripts was correlated with either decreased or increased mortality (increased or decreased LD_50_, respectively) across the 15 strains. Only Gene Ontology categories that overlap less than 50% and have p-values lower than 0.001 are shown. Circles that are larger and more shifted to the left indicate larger numbers of genes involved and stronger statistical signals, respectively. Categories related to either RNA metabolism or cytoplasmic ribosome assembly (left) or trans-membrane flux (right) are in shown black.

EMS is not only a mutagen, but also a toxin that can kill yeast cells without introducing lethal mutations [44]. To test whether the observed mortality can be linked to the toxic activity of EMS or to the rise of lethal mutation, we turned to functional analyses. We correlated the LD_50_ values with the abundance of 6207 mRNA transcripts reported for the same strains in a former study in which growth conditions were determined by low content of glucose and limiting concentration of phosphorous [34]. (We mimicked these conditions in our study). We then asked which Gene Ontology categories are overrepresented among transcripts best correlated (either positively or negatively) with LD_50._ This was done by calculating a Spearman’s correlation coefficient for each gene and ranking the list of all genes according to either decreasing or increasing coefficients followed by an analysis of overrepresentation of high ranks within GO categories [45]. After trimming the most overlapping gene categories [46], the results can be summarized in a way shown in Fig 3. Two conclusions emerged from these analyses. First, survival improved (LD_50_ values were high) with increasing expression of genes responsible for rRNA processing and biogenesis. Second, survival decreased (LD_50_ values were low) with increasing expression of genes coding for membrane-bound permeases and transporter proteins. The latter finding is a hint that high mortality following mutagenesis resulted from high susceptibility of the strains’ proteomes to EMS rather than from the rise of different numbers of lethal mutations in their genomes.

### Robustness of growth correlates positively with the expression of aerobic respiration genes

**Fig 4:**
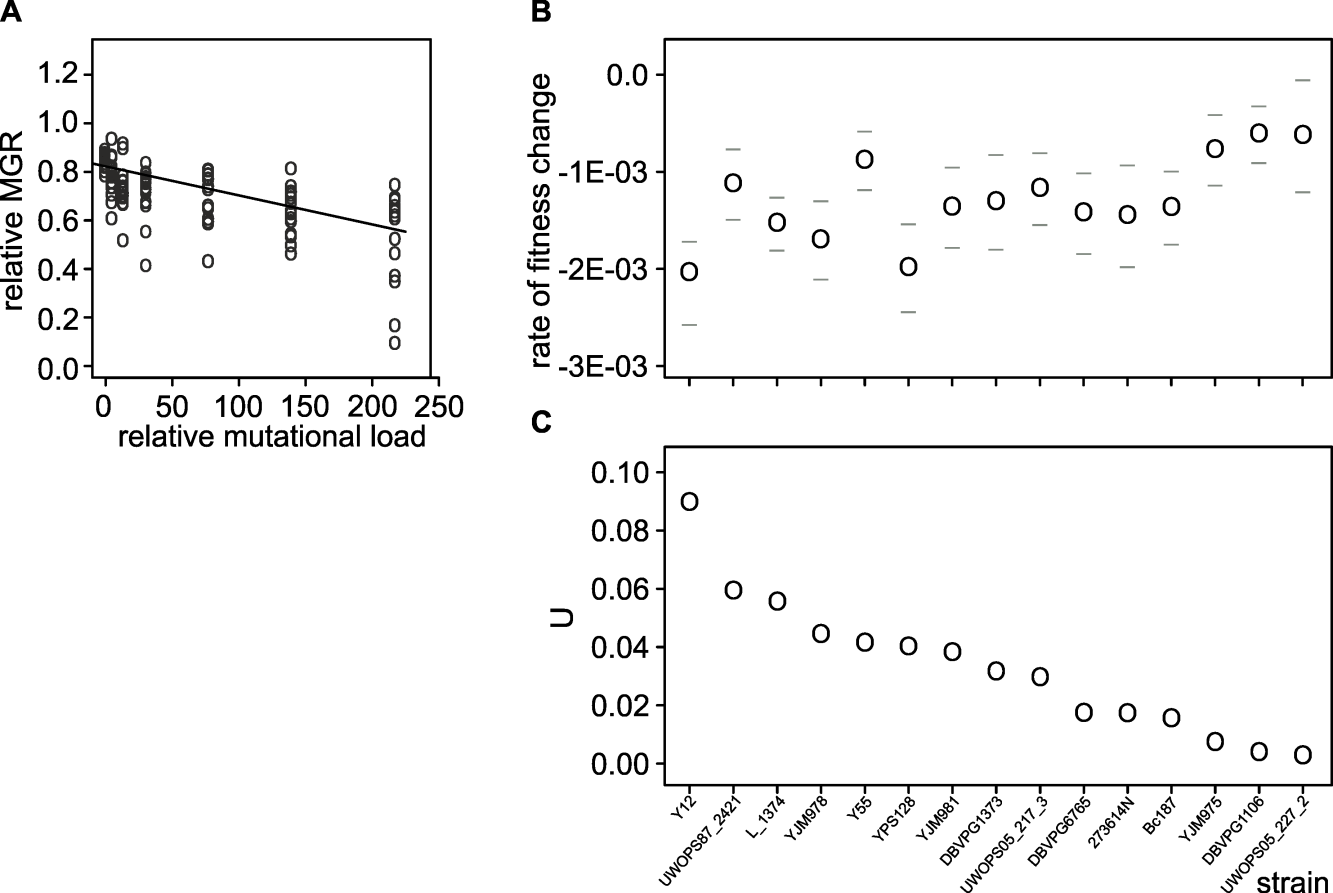
Genetic robustness estimated as the decline of the maximum growth rate (MGR) along increasing mutation load resulting from an earlier EMS treatment. (A) An example of a single strain’s declining MGR values (normalized by the MGR of untreated cells) related to the increasing relative mutation load (defined in Fig 1B). The slope of the line, *ΔM*, is the rate of fitness (MGR) decline with increasing number of mutations. (B) *ΔM* values with 95% confidence limits of individual strains (least robust have highest negative *ΔM* values). (C) *U*, the minimal number of mutations (with effect on MGR) obtained from the Bateman-Mukai equation. *U* values are ranked according to increasing robustness (least robust strains have highest *U*). The ranks used for *U* were adopted also for *ΔM* to show a negative relationship between the two parameters.

To test for genetic robustness against non-lethal mutations, here understood as the ability to retain a relatively high growth rate, we measured the maximum growth rate (MGR) of clones picked at random from survivors of every strain at every dose of EMS. MGR was measured in medium with a reduced level of glucose (0.5%) and limiting level of phosphorus identical to that used in the above mentioned gene expression study [34]. The authors of that study used chemostats running at a set dilution rate to minimize the effects of growth rate variation on gene expression. We used batch cultures at 23°C to obtain an average growth rate possibly closest to that realized in chemostats, without inducing cold stress [47]. Fig 4A shows relative growth rates of individual isolates declining as intensity of mutagenesis increased. The intensity of mutagenesis is expressed as a “relative mutation load”, which was calculated by regressing Uraˉ mutant counts on EMS doses, from 0 to 40 μl/ml. Therefore, the relative mutation load accounts for the relation between the concentration of EMS and the number of mutations incurred (see legend of Fig 1B). A simple measure of genetic robustness is the mean decrease in the maximum growth rate across the whole gradient of mutagenesis, *ΔM*. The so-called Bateman-Mukai technique [48] takes into account not only an increase in the mean but also variation and yields *U*, an estimated minimal number of phenotypically distinguishable mutations (negative growth effects). Fig 4B and 4C show that both *ΔM* and *U* vary extensively among strains and, as expected, are negatively correlated. In the following analyses, we use *U* as it is based both on the means and variances of the phenotypes of mutated strains and more appropriate to ask how often a damage is visible. (*U* can be interpreted as a proportion of molecular mutations with an effect on growth.) Returning to the question of the double effect of EMS, toxic and mutagenic, we note that mortality and growth impairment are not correlated (Pearsons’s correlation coefficients between LD_50_ and U_30_, *r* = 0.028, *n* = 15), *p*>0.05). Because the two traits react differently to EMS, they should depend on different cellular processes.

**Fig 5:**
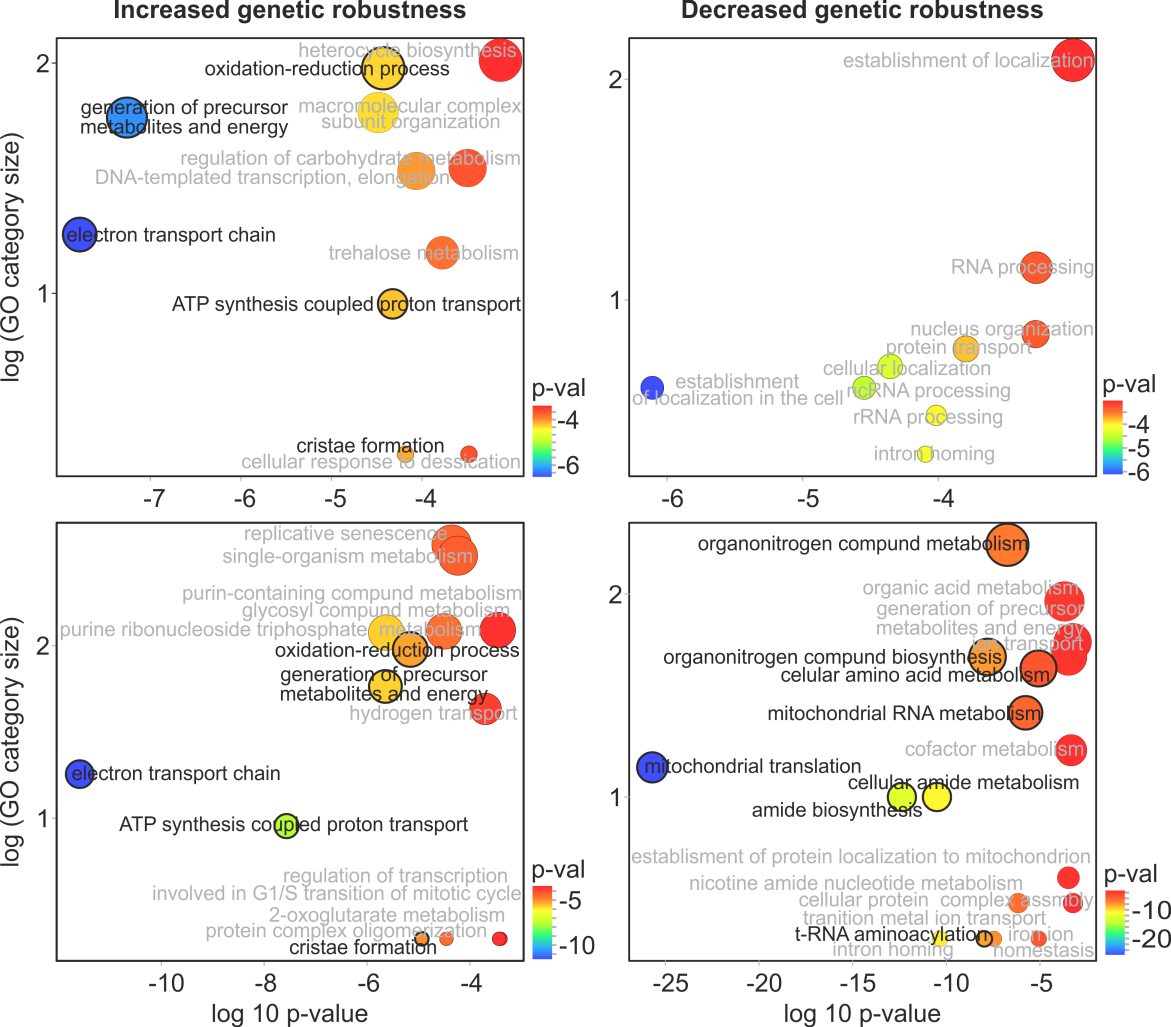
Test for functional determinants of growth of mutagenized strains (indicating robustness against non-lethal mutations). Enhanced expression of mRNA transcripts was correlated with either increased or decreased robustness (decreased or increased *U*, respectively). Upper panels refer to analyses done for the EMS range from 0 to 40 μl/ml (as in all other analyses reported here); lower panels refer to the EMS range from 0 to 30 μl/ml (thus relates to robustness in the face of lower mutation loads; note that results obtained with both ranges are generally similar). Only Gene Ontology categories which overlap by less than 50% and have p-values lower than 0.001 are shown. Circles that are larger and more shifted to the left indicate larger numbers of genes involved and stronger statistical signals, respectively. Categories related either to oxidative phosphorylation (right) or mitochondrial translation (left) are shown in black.

To test this assertion, we correlated *U* with the mRNA expression datasets in the same way as we previously did for LD_50_. The statistically strongest and functionally least overlapping results of the mRNA analysis are shown in Fig 5. The most remarkable finding was that the robustness was highest when expression of genes involved production of ATP on the electron transport chain was highest. When the analyses were restricted to no more than 30 μl/ml of EMS, a strong signal of an opposite relation was detected: robustness decreases when the mitochondrial translation intensifies (Fig 5B). In the discussion section, we develop an argument that the high efficiency of oxidative phosphorylation and low of mitochondrial translation are linked and beneficial for genetic robustness.

### Experimental verification of the dependence of genetic robustness on metabolic energy supply

**Fig 6:**
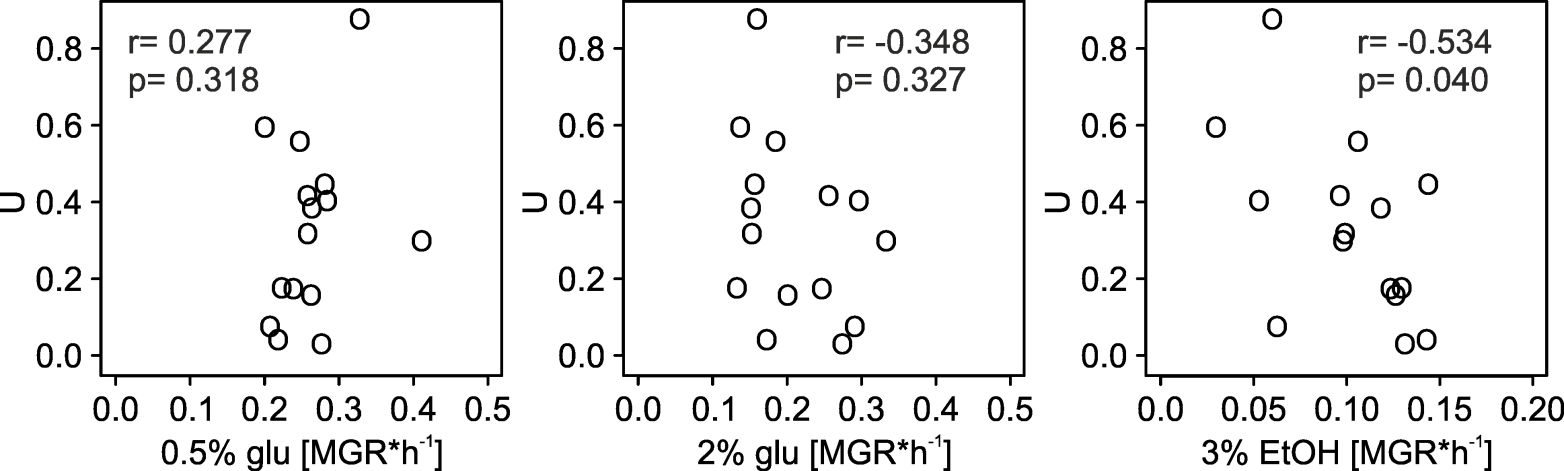
Robustness of the 15 strains in relation to performance on different energy sources (estimated as the maximum growth rate, MGR). Low values of *U* indicate high genetic robustness estimated under standard conditions for this experiment (0.5% glucose).

To verify whether robustness can be explained by energy metabolism, we performed three independent tests: growth on different carbon sources, growth after chemical inhibition of oxidative phosphorylation and growth after boosting oxidative phosphorylation through genetic manipulation. In the first test, we grew the collection of non-mutagenized strains on different carbon sources (23°C, P-limited medium with addition of either 0.5% glucose, 2% glucose or 3% ethanol). Fig 6 shows that strains that excelled in resisting the burden of mutations (lowest *U* in Fig 4C) were not among those growing well on 0.5% glucose (the medium used in the main experiment), nor on high glucose. It appears that mutational robustness did not result from the ability to adjust metabolism specifically to the environment used. Rather, it was correlated with the ability to grow well on 3% ethanol, in line with the results of the GO analysis.

**Fig 7:**
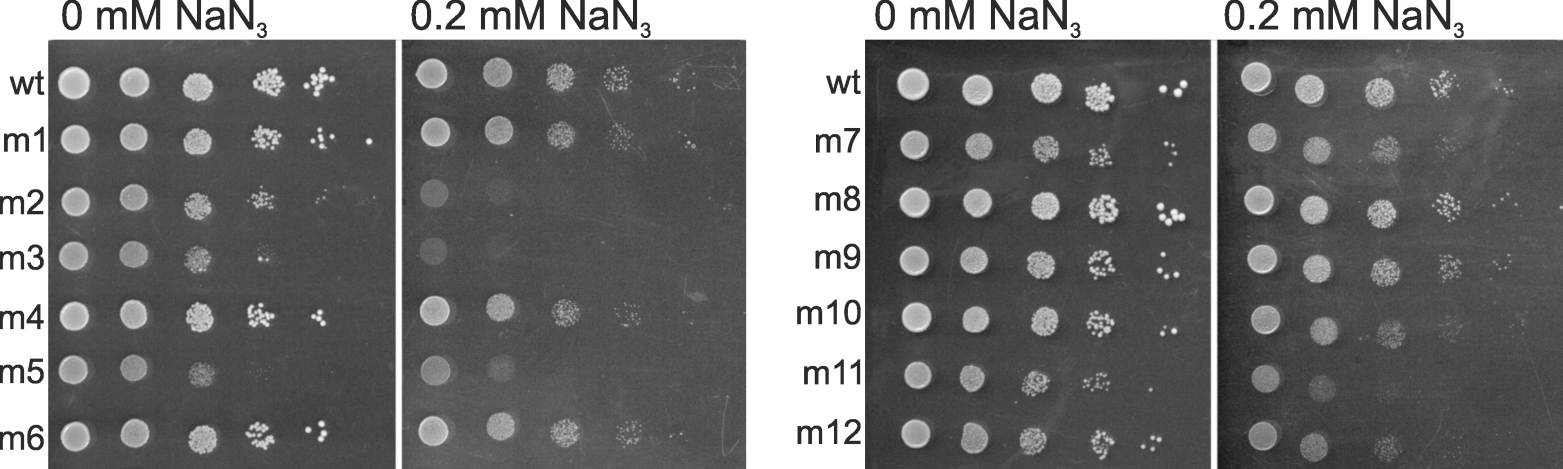
The impact of inhibition of the electron chain on growth. A non-mutated reference strain and a random sample of 12 EMS-induced mutants (all of the BY4742 background) were grown in the absence or presence of 0.2 mM sodium azide, which inhibits cytochrome c. Except for agar, growth conditions were the same as in the genetic robustness assays (see Fig 4).

In the second test, we sought to directly manipulate the profile of energy metabolism within one strain instead of using multiple strains differing in this respect. We treated a standard laboratory strain, BY4742 *MATα*, with 30 μl/ml doses of EMS in the same way as in the experiments described above. We then exposed a random sample of the resulting mutants to sodium azide (NaN_3_), which inhibits specifically the electron transport chain by binding to the cytochrome c oxidase complex. In this test, we applied the same temperature and medium, but we checked for growth on agar surface in a standard spotting assay. (We were unable to find a sufficiently long phase of stable exponential growth in liquid cultures with NaN_3_ added). We expected that moderate doses of NaN_3_ would hamper growth of mutagenized cells significantly stronger than that of non-mutagenized cells. Indeed, Fig 7 shows that mutants were often affected more than the non-mutagenized control.

**Fig 8:**
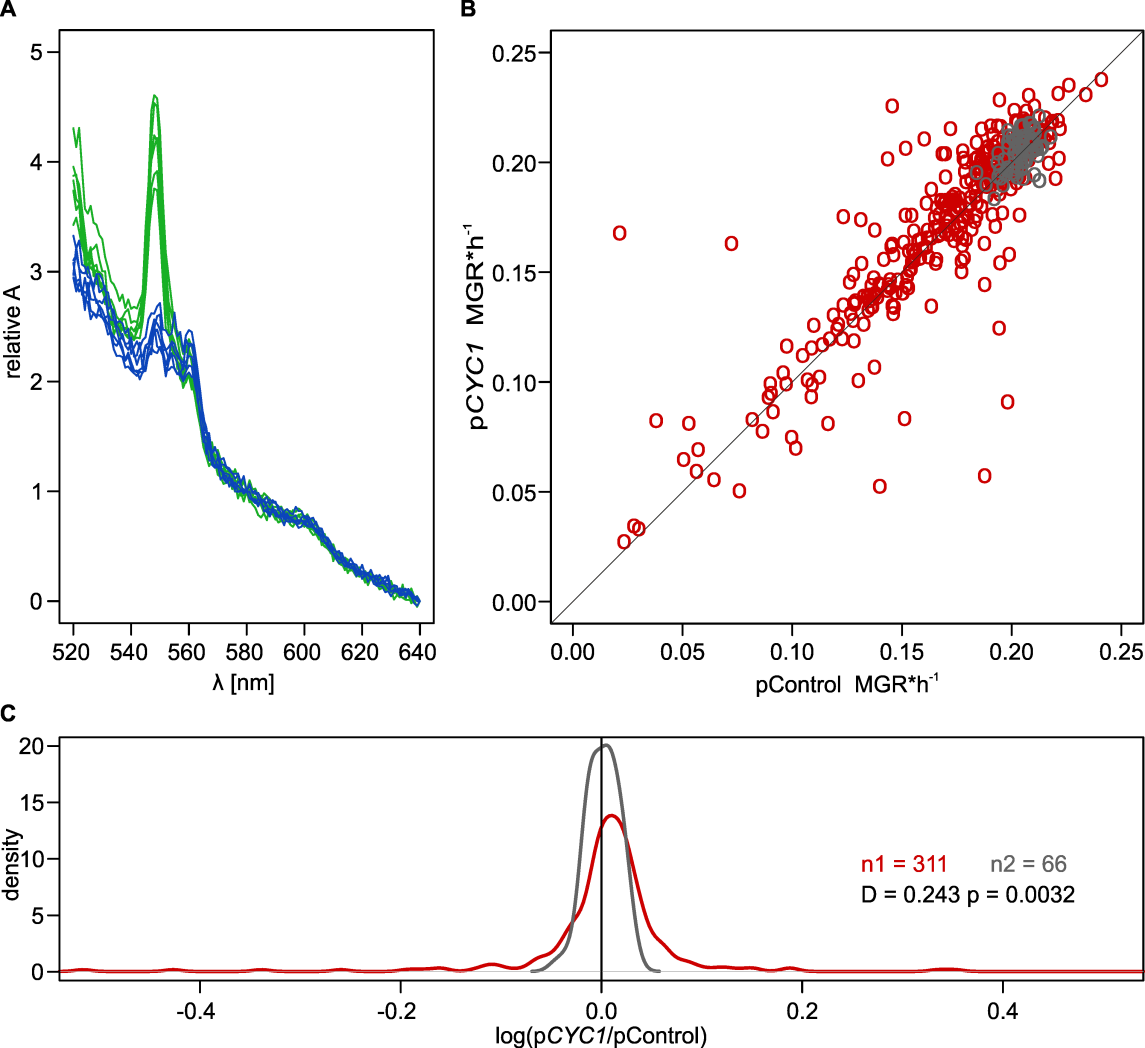
Effects of the cytochrome c coding gene (*CYC1*) overexpression. (A) Absorption spectra of BY4742 when overexpressing *CYC1* (green, 6 replicates) or not (blue, 6 replicates). Relative absorption was calculated by subtracting the absorption at 640 nm and dividing by the absorption at 580 nm within each replicate. The cellular level of the cytochrome c in its reduced form is indicated by the peak at 550 nm. The downstream enzyme (cytochrome c oxidase) peaks at 600 nm. (B) MGR of BY4742 transformed with either p*CYC1* or control plasmid (pControl, see Methods for details). Black circles denote replicate estimates for a non-mutagenized reference strain. Red circles denote individual mutated strains. Means of two replicates are used as coordinates. Results of ANOVA are in the main text. (C) Density plots of log p*CYC1*/pControl ratios of MGR for mutant (red) and reference (gray) strains. Statistics refer to results of a Kolgomorov-Smirnov test.

In the third test, we wanted to boost functioning of the oxidative phosphorylation pathway. The electron transport chain comprises several multi-protein complexes, and several genes would have to be overexpressed to elevate the activity of just one of them. The cytochrome c is outstanding in this respect, being composed of a heme group and a single polypeptide (Cyc1 in yeast). We transformed BY4742 with a multicopy plasmid carrying the *CYC1* gene under control of the *TEF* promoter and also a control plasmid, one of the same backbone and trophic markers, but with no gene overexpressed (see Materials). Fig 8A shows that overexpression of *CYC1* increased the cellular level of cytochrome c, especially in its reduced form, indicating that this step in the transportation of electrons was successfully upregulated.

We then obtained a large number of BY mutants (with 30 μl/ml of EMS). We transformed each of them with each of the two plasmids and measured MGR in the same way as in our previous assays. Results are presented in Fig 8B. There were 311 mutants that were successfully transformed with both plasmids, and each transformant was assayed twice. MGR differed greatly between mutants (*F* = 25.97, *dfs* = 310, 932; *p*<<0.0001). Most importantly for our hypothesis, there was a statistically significant difference in the mean MGR of mutants bearing either the p*CYC1* or control plasmid (respective means 0.1699 and 0.1668; *F* = 12.833; *dfs* = 1, 932; *p* = 3.58×10^−4^). To perform analogous tests for the non-mutated reference strain, we paired at random 66 replicate wild-type clones overexpressing p*CYC1* with 66 replicates expressing the control plasmid (each measured twice). There was some variation among pairs created in this way (*F* = 1.512; *dfs* = 65, 197; *p* = 1.63×10^−2^), which was likely introduced by assay block effects. Crucially, there was no difference between wild-type clones with p*CYC1* and control plasmid within the reference strain (respective means 0.2042 and 0.2034; *F* = 0.036; *dfs* = 1, 197; p = 0.849). The average advantage of mutants bearing p*CYC1* over those with the control plasmid did not result from strong but relatively few effects. Positive effects, measured as log(MGR_p*CYC1*_/MGR_pControl_) were widespread leading to a statistically significant shift in modal region of frequency distribution (Fig 8C). In sum, all three of our tests consistently support the notion that efficient energy metabolism yields genetic robustness in the 15 yeast strains we examined.

## Discussion

We studied genetic robustness in yeast by measuring the performance of a collection of diverse strains subject to mutagenesis. We found that mutagenesis resulted in considerably different phenotypic responses in different strains. There were differences in mortality and in the severity of growth defects among survivors. The two effects were statistically uncorrelated and associated with different patterns of gene expression. High mortality (high susceptibility to the toxin, low LD_50_) correlated with high expression of genes coding for membrane-bound transporter proteins and permeases. EMS is known to bind the thiol groups of proteins [44]. These groups are ubiquitous in the proteins residing in the cell membrane that are exposed to the external environment. There was also a strong correlation between low sensitivity to the toxin (high LD_50_ values) and high expression of genes responsible for rRNA processing and protein synthesis. This result is also understandable. To enable recovery after EMS treatment, cells were transferred to standard medium with a high concentration of glucose, which is a strong signal to start growth. Strains that were able to rapidly activate the translational machinery after mutagenesis had therefore a better chance of escaping the toxic effect of EMS-induced protein damage [49, 50]. We conclude that mortality appeared to result from the toxicity of EMS rather than from the introduced mutations.

The second of the studied traits, the ability to grow well despite carrying random mutations, is an evident sign of genetic robustness. Growth rates correlated positively with high expression of genes coding for oxidative phosphorylation and negatively with high expression of genes coding for mitochondrial ribosomes (Fig 5). Both oxidative phosphorylation and mitochondrial translation occur inside mitochondria. In our experiment, as in the former gene expression study, glucose was relatively low and phosphorous was limiting [34]. It has been shown that, for growth rates similar to those estimated here, raising the fraction of energy acquired through oxidation makes the metabolism substantially more efficient [51]. The observed requirement for low expression of the mitochondrial ribosome proteins fits this conjecture. In budding yeast, unlike other organisms, growth rate remains high even if the expression of mitochondrial ribosome proteins declines [52]. More importantly, there is only one RNA polymerase for all genes residing on the mitochondrial chromosome, including elements of the oxidative phosphorylation complexes and mitochondrial ribosomes [53]. It has been demonstrated that mitochondrial rRNAs can outnumber mitochondrial mRNAs coding for elements of oxidative phosphorylation complexes by a factor of 30 [54]. Therefore, even a small decrease in expression of ribosomal genes should result in substantially increased expression of the oxidative phosphorylation genes. In sum, the postulate that genetic robustness depends critically on the ability to generate metabolic energy through oxidative phosphorylation appears to be well founded, both in terms of statistical results and functional interpretation of our data.

Subsequent experiments supported our hypothesis. A simple test of growth on glucose and ethanol showed, that not proficiency in fermentation but in respiration correlated with genetic robustness (Fig 6). Both fermentation and respiration are used to generate ATP when glucose is relatively low and growth is slow. Respiration actually dominates under these conditions in the G_1_ phase when most of biosynthesis, and thus growth, is accomplished [55, 56]. The two other tests were based on experimental manipulation. They showed that growth rates of mutants decreased when the electron transport chain was inhibited chemically, and increased when one of its elements, cytochrome c, was overexpressed. Sodium azide is well known and often used as a specific inhibitor of the electron transport chain [57]. There is no comparably easy way to boost respiration. The electron transport chain is composed of several protein complexes. The only element that is critical for the chain and formed from a single polypeptide is cytochrome c, which was the reason why we chose to overexpress this particular protein. But, cytochrome c cooperates with two large complexes in transporting electrons, its reductase and oxidase, and requires assistance from several proteins to mature and end up in the intermembrane matrix of the mitochondrion. These proteins were not overproduced. We consider it remarkable that we nevertheless observed a significantly positive effect on fitness in most mutant clones. Some mutants appeared to suffer, but it is possible that supplying more energy to improper functions, such as altered signalling or transportation, can be harmful. In sum, the combined growth rate data obtained in this study, the expression data from a former study of the same strains [34] and literature on yeast metabolism, support the conclusion that the genetic robustness of yeast cells is critically dependent on the availability of energy. This result accords with a recent finding that the ability to generate additional ATPs through even a minor redirection of the carbon flux from fermentation towards respiration helps to ameliorate negative effects of production of unnecessary proteins [58].

There was no indication of a positive correlation between genetic robustness and high expression of molecular chaperones or other proteins that could potentially enhance genetic robustness. The GO classification contains both general and narrow categories of molecular chaperones, but none of them showed up as enriched, even if liberal criteria of statistical significance were applied. It is possible that refolding of destabilized proteins is most important under environmental stress. In our experiment, environmental stress was absent and proteins destabilized by mutations may have been too rare to incite a protective activity of chaperones [59–63]. Moreover, the expectation that chaperones typically buffer negative effects of mutations may not be universally true. We have postulated that the Hsp70 chaperones can rather help to dispose destabilized proteins instead of assisting their refolding. However, such proteins could be then at too low levels to fulfill their functions and therefore their negative effects would be increased [28, 29]. A recent study has demonstrated that the Hsp90 chaperone does act as a buffer for the effects of standing genetic variation, but it actually increases the effects of new random mutations [64]. A GO analysis should not be taken as evidence that certain gene categories, such as chaperones, are not important for the studied trait, because the method is effective in detecting only sufficiently strong positive signals. Nevertheless, we suggest that great care is needed when considering the role of molecular chaperone in masking of mutational damage.

The main conclusion emerging from our study is that genetic robustness partly rests on metabolic vigor. It implies that robustness can be aided by any feature of an organism helping its metabolism to function under the given environmental conditions. The efficiency of energy metabolism is probably one of general agents of robustness. It is possibly less critical when, for example, resources are abundant and growth depends mostly on the capabilities of assembling large numbers of ribosomes and sustaining their efficient functioning, which appear to be more challenging in terms of maintaining structures than securing energy [49, 65]. However, most microbial cells in the wild grow slowly or do not grow at all [66]. Effective energy metabolism is likely crucial under such circumstances, and especially so when some elements of the cell are damaged by mutations. Indeed, wild yeast strains bear sizable amounts of mutations, suggesting that natural selection is typically not effective enough to purge them [37]. The more complex systems are the more likely imperfections to occur. Over long time intervals, the ability to generate sufficient levels of energy could be the main force driving the evolution of eukaryotes, that is, making organisms complex, evolvable and robust [67].

## Materials and methods

### Strains and plasmids

A collection of yeast isolates originating from the wild and human-associated environments [33] has been converted into a set of strains that were stably haploid, auxotrophic for uracil and resistant to hygromycin B and geneticin, *ho*::*hph*MX4 *ura3*::*kan*MX4 [41]. From those, we used a subset of *S*. *cerevisiae* haploid strains in our former study [39]. These, and their *MATα* counterparts, were selected also for the present experiment, except for L-1528, DBVPG6044 and NCYC10, as they were not included into a study of gene expression which provided data used for our Gene Ontology analyses [34]. Except of being included in that study, there were some other constrains for strain selection. One was the suitability for reliable measurements of the maximum growth rate. The measurements had to be carried out in conditions similar to those used in a study in which data on gene expression had been collected [34]. UWOPS83-787.3 YPS 606 and SK1were dropped due to poor/undetectable growth or intense cell aggregation in the MGR assays described below. Another test of suitability was the assay or uracil proto/auxotrophy. A functional allele of the *URA3*gene derived from *Candida albicans*, inserted within the MX4 cassette on pAG60 plasmid, was used as a replacement for either *kan4*MX4 or *hph*MX4 residing on chromosomes. For each strain, *MATα URA3* and *MAT***a** *URA3* strains with alternative resistance markers left were obtained using PEG/LiAc transformation protocol [68]. Strain UWOPS03_227.2 was discarded because it typically yielded only a few colonies on the 5-FOA medium independent of the dose of EMS applied in the mutagenesis. As a result, 15 strains performed satisfactorily in both the 5-FOA and the maximum growth rate assays which permitted their use in the subsequent experiments. The final list of strains used in the mutagenesis and assays of genetic robustness comprises: L_1374, DBVPG1106, DBVPG1373, YJM975, YJM978, YJM981, DBVPG6765, BC187, 273614, YPS128, Y12, UWOPS87_2421, UWOPS05_227_2, UWOPS05_217_3, Y55.

In experiments following the assays of robustness we used BY4742 *MATα*, and two plasmids: pKATO1 *HIS3 leu2d* P_*TEF*_-*CYC1* (p*CYC1*) and pKATO2 *HIS3 leu2d* P_*GAL1*_-*URA3-YFP* (pControl). Both plasmids were derived from pRS425 (NCBI gi:416323). In all tests, plasmids were stabilized by omitting histidine in growth media which results in a relatively low number of plasmids per cell [69]. Galactose was never used and therefore expression of the fusion protein from the pControl plasmid was absent.

### EMS mutagenesis

Aliquots of 15 ml of stationary phase cultures, grown in SC-uracil at 30°C with 250 rpm shaking, were prepared for every strain and mating type. These were then equalized to OD = 1.2 and dispensed into microcentrifuge tubes, 1 ml per tube. Cells were centrifuged and the pellets were washed with 1 ml of potassium phosphate buffer (pH = 7) and then re-suspended in it. EMS was dispensed to set up a gradient of 0, 2, 5, 10, 15, 20, 25, 30, 40 and 60 μl/ml. (Not all of those concentrations were used in particular phenotypic assays, e.g. the highest EMS dose turned out to be too severe and was discarded). The mixtures were incubated for 1 hour at 30°C with periodic vortexing. They were then spun down, supernatant was decanted and samples were washed with 10% sodium thiosulfate (Na_2_S_2_O_3_), re-suspended in 1ml of water and left overnight at 4°C. The treated cells were then transferred to new growth medium and the new cultures were allowed to reach the stationary phase again. This allowed to complete several divisions and therefore fix the effects of EMS treatment. These new cultures were used to initiate screens with 5-FOA for Uraˉ phenotype.

### Cell mortality

To test the effects of EMS on survival, 0.1 ml samples of the overnight cultures (kept at 4°C, see above) were serially diluted and grown at 30°C for 2 or 3 days on YPD agar plates. The plates were then photographed and colonies were counted with OpenCFU software [70] or manually, if necessary. The obtained counts were used to plot survival curves and calculate the LD_50_ Δ with the *drm* function of the *drc* package in R.

### Mutant frequency

The remaining portions of the overnight 4°C cultures were transferred to 10 ml YPD and incubated at 30°C for 3 days. OD was measured (TECAN) and equalized to 1.6. Samples of the resulting cultures were plated onto synthetic complete plates supplemented with 0.1% of 5-FOA. After 3–4 days of incubation at 30°C, colonies were counted manually.

### Growth rate

The ‘cell mortality’ assay yielded colonies that developed from mutagenized cells on YPD plates. These colonies were selected at random and streaked to single cells to ensure that colonies derived this way were clonal. In sum, a total of 112 post-mutagenesis colonies of every strain (8 clones per every mating type, per 7 EMS doses: 0, 2, 5, 10, 20, 30,40 μl/ml) were was drawn at random, and after streaking to singles, one clone was derived for each of them. The resulting clones were grown individually as 200 μl micro-cultures in flat-bottom titration plates in order to measure MGR. The medium used in this assay was synthetic with low carbon (5 g/l of glucose) and limiting phosphorous levels (10 μg/l of sodium phosphate monobasic anhydrous—USP), as described previously [34].

### Number of phenotypic effects

The Bateman-Mukai formulae were used to estimate the number of negative growth effects for each strain [48]. The maximum average effect of a mutation is: *a = ΔV / 2ΔM*, the minimum number of effects is *U = 2(ΔM)*^*2*^ */ ΔV*, where *ΔM* is a decrease in the average *MGR* and *ΔV* is an increase in the variance of *MGR*. Averages (*M*s) and variances (*V*s) were calculated for 16 replicate clones per strain per EMS dose (the two mating types were polled as there was no statistical difference between them).

### Spectrophotometric analysis of the cytochrome c

Cultures were grown under conditions used in the assay of genetic robustness. Intact cells were harvested at the exponential growth phase, cooled, condensed to form 1 mm thick paste, and immersed in liquid nitrogen prior to measurement [71, 72].

## Supporting information

S1 Table Data referring to statistical analyses and graphs as identified by names of consecutive sheets. (XLSX)
